# Population Pharmacokinetics of *Cis*-, *Trans*-, and Total Cefprozil in Healthy Male Koreans

**DOI:** 10.3390/pharmaceutics11100531

**Published:** 2019-10-14

**Authors:** Ji-Hun Jang, Seung-Hyun Jeong, Hea-Young Cho, Yong-Bok Lee

**Affiliations:** 1 College of Pharmacy, Chonnam National University, 77 Yongbong-ro, Buk-Gu, Gwangju 61186, Korea; jangji0121@naver.com (J.-H.J.); rhdqn95@naver.com (S.-H.J.); 2 College of Pharmacy, CHA University, 335 Pangyo-ro, Bundang-gu, Seongnam-si, Gyeonggi-Do 13488, Korea; hycho@cha.ac.kr

**Keywords:** cefprozil, population pharmacokinetics, modeling, *cis*- and *trans*-isomers

## Abstract

Cefprozil, one of cephalosporin antibiotics, has been used extensively in clinics. However, pharmacokinetic (PK) information on cefprozil is still very limited. There have been no reports of population pharmacokinetics (PPKs). A PPK model for cefprozil will be a great advantage for clinical use. Thus, the aim of this study was to develop a PPK model for cefprozil for healthy male Koreans. Clinical PK and demographic data of healthy Korean males receiving cefprozil at a dose of 1000 mg were analyzed using Phoenix^®^ NLME™. A one-compartment model with first-order absorption with lag-time was constructed as a base model. The model was extended to include covariates that influenced between-subject variability. Creatinine clearance significantly influenced systemic clearance of cefprozil. The final PPK model for *cis*-, *trans*-, and total cefprozil was established and validated. PPK parameter values of *cis*- and total cefprozil were similar to each other, but different from those of *trans*-isomer. Herein, we describe the establishment of accurate PPK models of *cis*-, *trans*-, and total cefprozil for healthy male Koreans for the first time. It may be useful as a dosing algorithm for the general population. These results might also contribute to the development of stereoisomeric cefprozil.

## 1. Introduction

Cefprozil is a cephalosporin type drug that is administered orally. Its clinical effect as an antibiotic has long been confirmed. The range of antimicrobial activity of cefprozil is very broad. Its effect on resistant bacteria is also excellent. Thus, it is very important for clinical use. Wiseman et al. [1] have demonstrated the in vitro activity of cefprozil in various organisms. In particular, cefprozil has been reported to exhibit antibacterial activity against Gram-positive bacteria such as *Streptococcus pyogenes*, *Streptococcus pneumoniae*, *Streptococcus agalactiae*, and *Staphylococcus aureus*. Furthermore, it has been reported that cefprozil exhibits antibacterial activity against *Haemophilus influenzae*, *Moraxella catarrhalis*, *Neisseria gonorrhoeae*, many Enterobacteriaceae, and certain anaerobic organisms [1]. Broad antibacterial range and activities of cefprozil have been reported in the past [2,3,4]. The reason why cefprozil can exhibit such broad antibacterial range and activity against resistant bacteria is due to physicochemical properties of cefprozil, which is stable to hydrolysis by many beta-lactamase enzymes [1].

Cefprozil has been used clinically for the treatment of persistent or recurrent acute otitis media. It has also been used extensively in a variety of diseases related to skin infections and tonsillopharyngitis (including upper and lower respiratory tract infections) [5,6,7,8]. Unfortunately, pharmacokinetic (PK) information on cefprozil in humans is still very limited. There have been no reports of population pharmacokinetics (PPKs) for cefprozil.

Cefprozil has been reported to have similar side effects to other orally administered cephalosporin antibiotics. The most frequently reported side effects are diarrhea, nausea, vomiting, and redness associated with gastrointestinal and skin system [9]. Although the reported incidence of adverse effects of cefprozil is low in humans, appropriate dosing settings using a PPK model might be needed to reduce adverse effects of cefprozil and maximize its therapeutic effect. PPK modeling can enable individualized pharmacotherapy and effective dose setting by quantifying the diversity of drug concentrations among individuals in the population with a variety of related physiochemical factors. Cefprozil has been reported to have no significant change in PK parameters in patients with liver function problems compared to normal control subjects. Therefore, it does not require dose adjustment [10]. However, in patients with renal impairment, cefprozil has significant changes in PK parameters such as decreased clearance and increased half-life, compared to normal control subjects. Therefore, patients with renal insufficiency require dose adjustment [11]. We will quantitatively reflect creatinine clearance (CrCl), an index of renal function in people, in the final cefprozil PPK model in this study, suggesting that scientific dose setting is possible. The PPK model for cefprozil would be a great advantage for clinical applications in these aspects.

Interestingly, cefprozil exists as an isomeric mixture of *cis*- and *trans*- at a ratio of about 9:1 [12,13]. Therefore, we attempted to establish PPKs for total cefprozil as well as PPK for each of *cis*- and *trans*-isomers of cefprozil in this study. These *cis*- and *trans*-isomers of cefprozil differ in their antimicrobial activities. Antimicrobial activities of the two isomers against Gram-positive bacteria are similar to each other. However, for Gram-negative bacteria, *cis*-cefprozil has six times greater activity than *trans*-cefprozil [9]. Therefore, the PPK for each of these isomers of cefprozil will be meaningful and interesting.

Therefore, the aim of this study was to develop a PPK model of cefprozil in healthy Korean adults. The developed PPK model is expected to be useful for establishing an effective dosing algorithm of cefprozil in healthy Korean subjects. It is also useful for providing information about the development of stereoisomerics.

## 2. Materials and Methods 

### 2.1. Study Design 

A total of 35 healthy Korean males from a bioequivalence study of cefprozil were included in this analysis. Each subject was physically normal without previous history of illness or hypersensitivity to any drugs. Subjects were excluded from this study if they were taking any medications, alcohol, or other drugs for at least one week prior to this study and throughout the study period. The study protocol was approved by Institutional Review Board of the Institute of Bioequivalence and Bridging Study, Chonnam National University, Gwangju, Republic of Korea (Bioequivalence Test No. 611; 11.06.2007). It was conducted in accordance with the revised declaration of Helsinki for biomedical research involving human subjects and rules of good clinical practice. All subjects provided informed written consent to undergo PK and bioequivalence studies. Bioequivalence studies were conducted as single-dose, randomized, two-way, open-label, and crossover studies. Only data from reference formulation were used for this analysis. After an overnight fast, subjects in each study group received a single oral dose (1000 mg) of cefprozil with 240 mL of water. Blood samples (8 mL) were collected into Vacutainer^®^ tubes (Becton–Dickinson and Company, Franklin Lakes, NJ, USA) before administration (0 h) and at 0.5, 0.75, 1, 1.25, 1.5, 1.75, 2, 3, 4, 8, and 12 h after oral administration. Following centrifugation at 5000× *g* for 20 min, plasma samples were transferred to polyethylene tubes and stored at −80 °C until analysis. After a washout period of seven days, each study was repeated in the same manner to complete the crossover design.

### 2.2. Determination of Plasma Cefprozil Concentrations

Plasma concentrations of *cis*- and *trans*-cefprozil isomers were determined using a validated ultraperformance liquid chromatography-electrospray ionization-tandem mass spectrometer (UPLC-ESI-MS/MS) method as described in a previous study [14]. Briefly, 10 μL of internal standard (IS, cefaclor, 500 ng/mL in plasma) and 1000 μL of methanol-3% formic acid in ethyl acetate (60/40, *v*/*v*) were added to 100 μL of plasma sample. After vortex-mixing for 5 min, the sample was centrifuged at 13,000× *g* for 5 min. Then 1000 μL of the supernatant organic layer was dried gently with a centrifugal vacuum evaporator under nitrogen gas at 40 °C for 3 h. The dried matter was reconstituted with 50 μL of mobile phase solution and vortexed for 5 min. After centrifugation at 13,000× *g* for 5 min, 5 μL of the supernatant (aliquot) was injected into the UPLC-ESI-MS/MS system. Plasma concentrations of total cefprozil were determined by the sum of plasma concentrations of *cis*- and *trans*-isomers.

The UPLC-ESI-MS/MS system consisted of a Shimadzu Nexera-X2 Series UPLC system (Shimadzu, Kyoto, Japan) coupled with a Shimadzu-8040 mass spectrometer (Shimadzu, Kyoto, Japan) with a DGU-20A degassing unit and an SIL-30AC autosampler. Optimized chromatographic separation of cefprozil isomers was conducted with a HALO-C_18_ column (100 × 2.1 mm i.d., 2.7 μm particle size; Advanced Materials Technology Inc., Wilmington, DE, USA) at an oven temperature of 40 °C. The mobile phase consisted of 0.5% formic acid in water containing 5% (*v*/*v*) of 5 mM ammonium formate (pH 3.0) buffer (mobile phase A; pH 2.0) and methanol (mobile phase B). Analysis was performed with a gradient elution and a flow rate of 0.3 mL/min. The elution program was as follows: 0–0.5 min (5% B), 0.5–2.0 min (5–60% B), 2.0-3.2 min (60% B), 3.21–4.0 min (5% B). All analytical procedures were evaluated with negative electrospray ionization. Quantification was achieved using multiple reaction monitoring (MRM) modes at *m*/*z* 388.00 → 249.20 for *cis*-, *trans*-cefprozil and *m*/*z* 365.90 → 286.20 for IS. Acquisition and analysis of data were achieved using a LabSolutions program with collision energy of 13 and 21 eV for cefprozil (*cis*- or *trans*-) and IS, respectively. The injection volume was 5 μL.

### 2.3. Model Development

PPK analysis was performed using nonlinear mixed-effects model (NLME) approach (Phoenix^®^ version 8.1, Certara Inc., St. Louis, MO, USA). The first-order conditional estimates method with extended least squares estimation was used for PPK model development.

Basic PK parameters used in this analysis were clearance for the central compartment (Cl), volume of distribution for the central compartment (V), oral absorption rate constant (K_a_), and absorption lag time (T_lag_). Parameters obtained from noncompartmental and classic compartmental models were used as initial estimates. 

One or two compartment models with first-order absorption and elimination with or without absorption lag were tested to determine the structural base model. Model selection was based on statistical significance between models using twice the negative log likelihood (-2LL), Akaike information criterion (AIC), and goodness-of-fit plots.

Inter-individual variability (IIV) in PK parameters of *cis*-, *trans*-, and total cefprozil were explained using exponential error models as shown in the following equation:

*P_i_* = *P_tv_*·exp(*ŋ_i_*) (1)

where *P_i_* was the parameter value of the *i*th individual, *P_tv_* was the typical value of the population parameter, and *ŋ_i_* was the random variable for the *i*th individual which was normally distributed with mean 0 and variance ω^2^. 

The residual variability was evaluated with additive error on log transformed data (Equation (2)) or proportional error (Equation (3)) models:

*C_obs,ij_* = *C_pred,ij_*·exp(*ε_ij_*) (2)

*C_obs,ij_* = *C_pred,ij_*·(1 + *ε_ij_*) (3)

where *C_obs,ij_* and *C_pred,ij_* were the *j*th observed and predicted concentrations in the *i*th subject, respectively, *ε_ij_* was the intra-subject variability with mean 0 and variance σ^2^.

Candidate covariates were age, body weight, body surface area (BSA), total proteins, albumin, alanine aminotransferase (ALT), aspartate aminotransferase (AST), alkaline phosphatase (ALP), total bilirubin, blood urea nitrogen (BUN), creatinine (Cr), and creatinine clearance (CrCl). BSA was calculated based on Mosteller equation [15]. CrCl was estimated using the Cockcroft–Gault formula [16]. These candidate covariates were plotted against individual posthoc parameters to evaluate correlations between PK parameters and covariates. Continuous covariates were normalized by median values. Effects of each covariate were evaluated using additive, exponential, or power functions. Covariates were included by stepwise forward selection and backward elimination procedure. The inclusion of covariates was determined by change in objective function value (OFV). In forward selection procedure, covariates with decrease in OFV of more than 3.84 (*p* < 0.05) were remained in the base model. During backward elimination, covariates with a change in the OFV of more than 6.63 (*p* < 0.01) remained in the model.

### 2.4. Model Evaluation

Final established models were evaluated and verified both numerically and visually. Goodness-of-fit plots (including distribution of residuals), bootstrapping methods, and visual predictive check (VPC) were used to evaluate these models. Goodness-of-fit was evaluated by using diagnostic scatter plots as follows: (a) observed (DV) versus population predicted concentrations (PRED); (b) DV versus individual predicted concentrations (IPRED); (c) conditional weighted residuals (CWRES) versus time (IVAR); (d) CWRES versus PRED; and (e) quantile-quantile plot of components of CWRES.

The stability of final model was evaluated using the non-parametric bootstrap analysis. Repeated random sampling with replacement from the original data set generated 1000 replicates. Values of estimated parameters such as medians and standard errors from the bootstrap procedure were compared with those estimated from the original dataset. 

VPCs of the final established models were done using the VPC option of Phoenix^®^ NLME™. DV concentration-time data were graphically superimposed on median values and the 5th and 95th percentiles of the simulated concentration-time profiles. The model was expected to be precise if DV concentration data were approximately distributed within the 5th and 95th prediction interval (PI).

## 3. Results

### 3.1. Subjects Characteristics 

Bioequivalence data collected from 35 healthy Korean male subjects were included in this analysis. A total of 420 plasma samples were obtained for each PPK analysis of *cis*-, *trans*-, and total cefprozil. Age and body weight of subjects ranged from 21 to 27 years (24 ± 1.53 years, mean ± SD) and 53.1 to 91.8 kg (69.7 ± 10.0 kg, mean ± SD), respectively. Information for age, bodyweight, and biochemical parameters were complete for each participant. Detailed demographic characteristics of the population are summarized in Table 1.

### 3.2. Determination of Plasma Cefprozil Concentrations

Plasma concentrations of *cis*- and *trans*-cefprozil after oral administration at dose of 1000 mg were determined with an UPLC-ESI-MS/MS method as mentioned in Section 2.2. This method could simultaneously analyze both *cis*- and *trans*-isomers at a run time of 4 min per sample, enabling efficient PK studies on cefprozil. Linearities of calibration curves for *cis*- and *trans*-isomers of cefprozil were excellent in human plasma, ranging from 0.005 to 20 μg/mL and from 0.015 to 2.5 μg/mL, respectively. In addition, lower limits of quantitation for *cis*- and *trans*-isomers were very low (5 and 15 ng/mL, respectively). They were sufficient for PK studies after oral administration of cefprozil tablets to humans. Intra-batch accuracies for *cis*-cefprozil and *trans*-cefprozil ranged from 96.17% to 104.58% with precision (coefficient of variation; CV) <6.65%. Inter-batch accuracies for *cis*-cefprozil and *trans*-cefprozil by both methods ranged from 98.32% to 103.67% with precision (CV) <6.46%.

### 3.3. Model Development

PKs of *cis*- and total cefprozil were best described by the one compartment model with first order absorption, elimination, and an absorption lag. Two-compartment model did not provide a better fit than the one compartment model. Exponential model was used to describe IIVs on parameters V, Cl, and T_lag_. No random effect was considered for K_a_. Residual variability was explained using an additive error model in log-transformed data. Plasma concentrations of *trans*-cefprozil were also best fitted by the one compartment model with first order absorption and lag time. IIVs on Cl, T_lag_, and K_a_ in the base model were modelled using exponential model. IIV on V was not considered. Proportional model was selected as residual error model. A summary of base model development steps is shown in Table 2.

**Figure 1:**
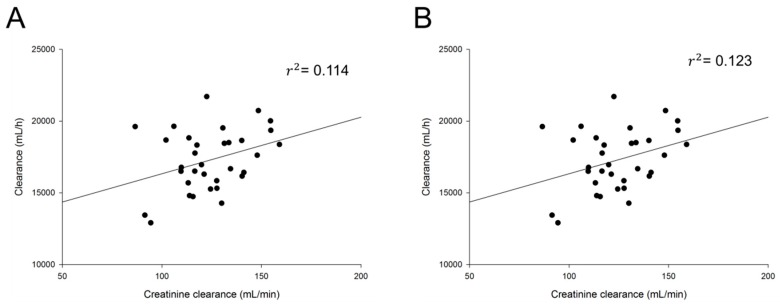
Relationship between individual predicted pharmacokinetic parameters and subject‘s characteristics. (**A**) Clearance of total cefprozil according to creatinine clearance; (**B**) Clearance of *cis*-cefprozil according to creatinine clearance.

Based on graphical exploration between PK parameters and covariates, final potential covariates were selected. The influence of each selected covariate was evaluated in the model. Table 3 summarizes the covariate selection process according to OFV. There was a significant correlation between CrCl and Cl of *cis*- and total cefprozil. The inclusion of CrCl in the PPK model for *cis-* and total cefprozil decreased OFV by 7.668 and 7.331, respectively (*p* < 0.05). Other covariates including weight, BSA, total protein and albumin failed to show significant influence on the models. On the other hand, no covariate affected PK parameters of *trans*-cefprozil. Figure 1 presents relations between selected covariates and Cl of *cis-* and total cefprozil.

The final models of *cis*-, *trans*-, and total cefprozil reflecting effects of covariates are described as follows:

***Cis*-cefprozil**

V = V*_tv_*·exp(*ŋ*_V_)

Cl = Cl*_tv_*·(1+(CrCl-124.41)^dCldCrCl^)·exp(*ŋ*_Cl_)

T_lag_ =T_lag *tv*_·exp(*ŋ*_Tlag_)

Ka = Ka*_tv_*

***Trans*-cefprozil**

V = V*_tv_*

Cl = Cl*_tv_*·exp(*ŋ*_Cl_)

T_lag_ =T_lag,*tv*_·exp(*ŋ*_Tlag_)

Ka = Ka*_tv_*·exp(*ŋ*_Ka_)

**Total cefprozil**

V = V*_tv_*·exp(*ŋ*_V_)

Cl = Cl*_tv_*·(1+(CrCl-124.41)^dCldCrCl^)·exp(*ŋ*_Cl_)

T_lag_ =T_lag,*tv*_·exp(*ŋ*_Tlag_)

Ka = Ka*_tv_*

Population estimates of *cis*-, *trans*-, and total cefprozil were 14,308.10, 34,617.50, and 14,713.10 mL for V and 17,150.70, 17,701.80, and 17,226.20 mL/h for Cl, respectively. Relative standard error (RSE%) was 1.28–56.16% in the final models. Eta shrinkage values of estimated PK parameters were considered acceptable (7.08–21.39%). In comparison with the base model, the final model with CrCl effect showed 1.15 and 1.04% reduction in the IIV for Cl of *cis*- and total cefprozil, respectively. Parameter estimates of the base model and final PPK model are listed in Table 4.

### 3.4. Model Evaluation

**Figure 2:**
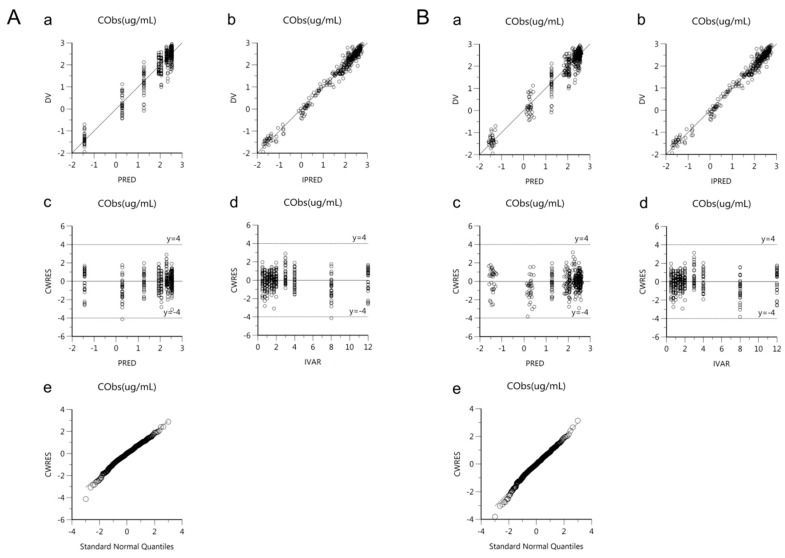
Goodness-of-fit plots of base model (**A**) and final model (**B**) for *cis*-cefprozil. (**a**) Observed plasma concentrations (DV) against population predicted concentrations (PRED); (**b**) Observed plasma concentrations (DV) against individual predicted concentrations (IPRED); (**c**) Conditional weighted residuals (CWRES) against population predicted concentration (PRED); (**d**) Conditional weighted residuals (CWRES) against time (IVAR); (**e**) Quantile-quantile plot of components of conditional weighted residuals.

**Figure 3:**
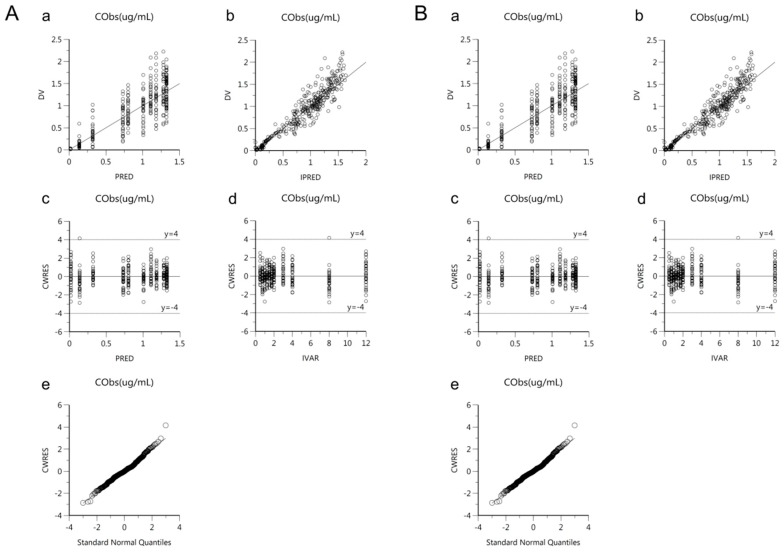
Goodness-of-fit plots of base model (**A**) and final model (**B**) for *trans*-cefprozil. (**a**) Observed plasma concentrations (DV) against population predicted concentrations (PRED); (**b**) Observed plasma concentrations (DV) against individual predicted concentrations (IPRED); (**c**) Conditional weighted residuals (CWRES) against population predicted concentration (PRED); (**d**) Conditional weighted residuals (CWRES) against time (IVAR); (**e**) Quantile-quantile plot of components of conditional weighted residuals.

**Figure 4:**
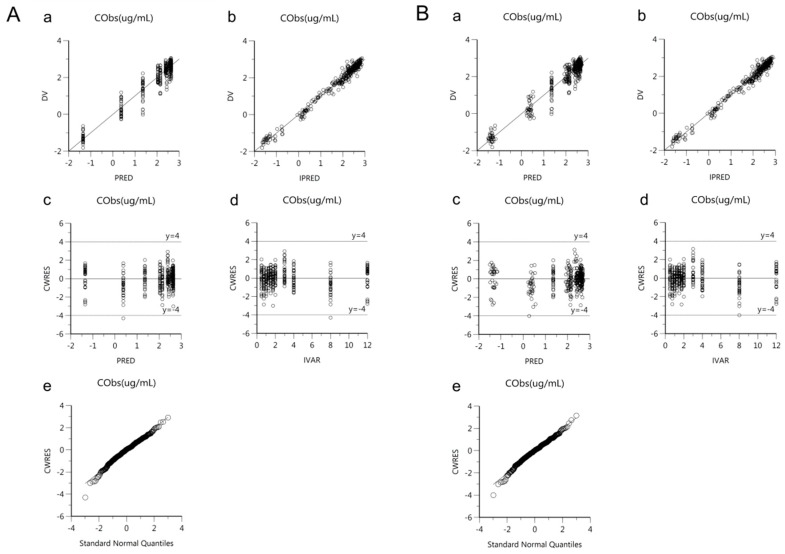
Goodness-of-fit plots of base model (**A**) and final model (**B**) for total cefprozil. (**a**) Observed plasma concentrations (DV) against population predicted concentrations (PRED); (**b**) Observed plasma concentrations (DV) against individual predicted concentrations (IPRED); (**c**) Conditional weighted residuals (CWRES) against population predicted concentration (PRED); (**d**) Conditional weighted residuals (CWRES) against time (IVAR); (**e**) Quantile-quantile plot of components of conditional weighted residuals.

Goodness-of-fit plots of the base and final models for *cis*-, *trans*-, and total cefprozil are shown in Figure 2, Figure 3 and Figure 4. Observed and predicted concentrations of *cis*-cefprozil were very consistent in the final model (Figure 2). CWRES were well distributed around zero. Residuals were also improved in the final model compared to the base model. As shown in Figure 3, predictions were generally fitted with observed plasma concentrations of *trans*-cefprozil. CWRES were distributed within 4, except for one point. In addition, the final model resulted in a good fit for plasma concentrations of total cefprozil (Figure 4). Observed and predicted concentrations fitted relatively symmetrical. CWRES were randomly distributed without any specific bias. Residuals greater than 4 in the base model showed some reduction in the final model. 

Bootstrap analysis demonstrated the robustness of the final PPK models for *cis*-, *trans*-, and total cefprozil (Table 5). Final parameter estimates were within the 95% CI range of bootstrap results, similar to median values of replicates (*n* = 1000).

**Figure 5:**
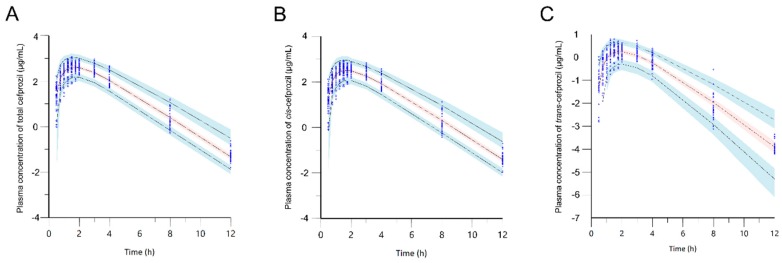
Visual predictive check of the final model for total cefprozil (**A**), *cis*-cefprozil (**B**), and *trans*-cefprozil (**C**). Dots indicate observed concentrations. Black dashed lines represent 5th, 50th, and 95th percentiles of the predicted concentrations; Blue shaded regions are 95% confidence intervals for predicted 5th and 95th percentiles. Red shaded regions are 95% confidence intervals for the predicted 50th percentiles.

Figure 5 shows VPC simulation plots for final PPK models of *cis*-, *trans*-, and total cefprozil. Most of these observed data were distributed within the 90% PI of the predicted value. These results proved the precision of the final models.

## 4. Discussion

Cefprozil is a class of cephalosporin antibiotics widely used in clinical practice. However, reported PK information for cefprozil is very limited. The reason for the lack of cefprozil PK studies seems to be that cefprozil has relatively mild side effects compared with antibiotics such as vancomycin or aminoglycosides that may cause serious toxicity. Nevertheless, it is necessary to determine PK characteristics of drugs and set individualized dose and usage to improve therapeutic effect and resistance of antibiotics. Several studies have reported that cefprozil may require clinical dose adjustment for some patient groups [11,17,18]. However, no studies have provided an accurate experimental basis. Therefore, this study aimed to establish PK model of cefprozil and identify factors that might affect PK characteristics based on data from a healthy Korean male population.

In this study, PKs of *cis*-, *trans*-, and total cefprozil were modelled as one compartment model with first order absorption and lag time. To determine PK characteristics of cefprozil and factors that might influence the variability of parameters, various error models and effects of various covariates were assessed. Goodness-of-fit plots for *cis*-, *trans*-, and total cefprozil demonstrated acceptable prediction of the final PPK models. Almost all CWRES were distributed within the range of ± 4 except for one data point in the model of *trans*-cefprozil. In addition, VPC simulations and bootstrap replicates demonstrated stability and accuracy of the final models. 

Only CrCl was found to influence the clearance of cefprozil. Since cephalosporins are mainly removed by renal elimination [19,20], renal function marker CrCl is expected to be a major covariate. The established PPK model demonstrated the relationship between CrCl and Cl. As shown in Figure 1, CrCl and Cl had a positive correlation, consistent with previous reports of PPK studies on other cephalosporin drugs [21,22,23]. For patients with renal insufficiency, a dosage reduction of cephalosporins including cefprozil has been recommended. Shyu et al. [24] have reported that a reduction in dosage is recommended in patients with CrCl of 30 mL/min or less. However, their report did not provide any specific basis on how much dose should be controlled. In our study, the relationship between CrCl and Cl was expressed by the equation using PPK model. It was found that a dose reduction was required for people with low CrCl. Our results showed that the IIV reduction on Cl with CrCl was 1.05–1.15%. Although the effect was small, it was confirmed that renal function clearly affected PKs of cefprozil in even a very limited change in CrCl obtained from healthy adult males. The contribution of this change is expected to be very significant in a wide range of CrCl changes in patients with renal impairment. In other words, our results suggest the possibility of setting accurate dose reflecting patient’s renal function (individual CrCl). On the other hand, liver function index AST, ALT, or ALP showed no significant effect on PK parameters. Our study supports results of previous reports showing that dose adjustment is not required depending on liver function [10,25,26]. In addition, there were no additional covariates that affected IIV on any parameters.

Unlike *cis*- or total cefprozil, PK parameters of *trans*-cefprozil were not explained by any covariate. Overall, characteristics of *cis*- and total cefprozil models were similar to each other. Such similar PPK and covariate effects of *cis*- and total cefprozil were attributed to the fact that *cis*-isomer accounted for 90% of total cefprozil. Therefore, characteristics of *cis*-isomer appear to be prominent in the profile of total cefprozil. Our study found that *trans*-cefprozil was not affected by covariate such as renal or hepatic function. These results indicate that administration dose of *trans*-cefprozil could be easily set when cefprozil is administered to patients with renal or hepatic impairment. This may provide important information for the development of formulation on *trans*-isomer of cefprozil in the future. 

Our study suggests that there might be a difference in the elimination of *cis*- and *trans*-isomer. Additional researches such as the analysis of cefprozil in urine samples should be conducted to clearly identify the elimination of the two isomers. In addition, there were differences in PK parameters of *cis*- and *trans*-cefprozil. To understand the clear reason and pathway, further studies on the in vivo kinetics of cefprozil is needed. 

Unexplained variability remained in V (ω; 35.6% in *cis*-isomer, 35.3% in total cefprozil) and lag time (ω; 36.1% in *trans*-isomer) (Table 4). Our study did not find any factor that could explain IIV for V or lag time. The information used in our study was insufficient to explain the remained variability. There might be another relevant covariate not detected yet. Intestinal absorption of oral beta-lactam antibiotics is known to be affected by intestinal peptide transporter 1 (PEPT1) [27,28]. In addition, other transporters might affect the absorption, metabolism and excretion of beta-lactam antibiotics, including cefprozil [29,30]. Further research using information on genetic polymorphisms including transporter type is needed to identify covariates that affect the variability of PK parameters that cannot be explained in this study. Additive residual variabilities were 0.193 and 0.189 μg/mL for *cis*- and total cefprozil, respectively. Proportional residual variability was 23.2% for *trans*-cefprozil. These residual errors could be derived from intra-subject variability, assay error, and model misspecification. 

Our model showed that there was no random variability on the absorption rate constant of *cis*- and total cefprozil. *Cis*-cefprozil was absorbed quickly within 2 h after administration. This pattern was similar in all subjects without any significant difference. Therefore, there was no significant difference in absorption rate constant of *cis*-cefprozil among individuals. The absorption pattern of total cefprozil was also similar to *cis*-isomer. This is considered to be due to dose similarity as mentioned above. On the other hand, PPK of *trans*-cefprozil did not include random effect on V. This was thought to be due to difference in physicochemical properties between *cis*- and *trans*-isomers. In general, factors affecting V exist in various ways such as physicochemical properties of drugs in addition to physiological factors including age and gender [31]. *Cis-* and *trans*-isomers differ in polarity which might have caused a difference in the V between these isomers.

Our PK parameters obtained by noncompartmental analysis were similar to the previously reported values. The reported maximum blood concentration (C_max_), time to reach C_max_ (T_max_) and area under the plasma concentration-time curve (AUC) of *cis*-cefprozil ranged from 12.3 to 17.3 μg/mL, 1.75 to 2.06 h, and 46.0 to 65.0 μg·h/mL, which is similar to our results of 15.0 μg/mL, 1.96 h, and 59.6 μg·h/mL. The half-life (t_1/2_) of *cis*-isomer in this study was 1.67 h, which was not significantly different from the reported values (1.72-1.87 h) [13,24,32]. In case of *trans*-cefprozil, the reported C_max_, T_max_, AUC, and t_1/2_ were 1.33–1.93 μg/mL, 2–2.13 h, 4.40–7.12 μg·h/mL, and 1.4–1.66 h, which were similar to our results (1.63 μg/mL, 1.96 h, 6.38 μg·h/mL, and 1.50 h) [13,32]. In addition, PK parameters of total cefprozil (16.6 μg/mL for C_max_, 2.11 for T_max_, 66.0 μg·h/mL for AUC, and 1.66 h for t_1/2_) in this study also did not differ significantly from the reported values for C_max_ (18.3–18.8 μg/mL), T_max_ (0.90–2.06 h), AUC (61.2–71.8 μg·h/mL), and t_1/2_ (1.20–1.69 h) [11,19,32].

Although our results described characteristics on PPKs of cefprozil, this study also had some limitations. First, our study was conducted with a limited group. Subjects were only healthy males aged from 21 to 27 years. Thus, our results cannot be completely generalized to clinical patients. As mentioned above, there was a lack of genetic information that might have influenced PK characteristics of cefprozil. To improve these limitations, further PPK analysis should be conducted with patients having on various conditions.

In summary, this is the first report to describe PPKs of cefprozil using plasma concentration data from healthy Korean males. The established PPK model in our study suggested that administration dose of cefprozil could be adjusted to reflect individual CrCl. Our results are expected to be useful as scientific basis for clinical use of cefprozil, and the development of formulation on *trans*-cefprozil for reanl or hepatic impaired patients in the future.

## 5. Conclusions

In this study, PPK models of *cis*-, *trans*-, and total cefprozil were developed using data in healthy Korean males. Plasma concentrations of *cis*-, *trans*-, and total cefprozil were well described by a one compartment model with first-order absorption and lag time. CrCl had significant effect on clearance of *cis*- and total cefprozil, while no covariate was found to be associated with PK parameters of *trans*-cefprozil.

## Figures and Tables

**Table 1:** Demographical data of subjects (*n* = 35).

Characteristic	Median (Min, Max)	Mean ± SD
Age (year)	24 (21, 27)	24 ± 1.53
Weight (kg)	69.5 (53.1, 91.8)	69.77 ± 9.99
BSA (m^2^)	1.84 (1.61, 2.16)	1.84 ± 0.16
Total proteins (g/dL)	7.5 (6.8, 8.4)	7.48 ± 0.37
Albumin (g/dL)	4.9 (4.4, 5.3)	4.84 ± 0.25
AST (U/L)	18 (12, 38)	20.31 ± 5.70
ALT (U/L)	17 (10, 42)	19.63 ± 8.36
ALP (U/L)	66 (46, 105)	69.83 ± 15.63
Total bilirubin (mg/dL)	0.92 (0.31, 2.23)	1.01 ± 0.39
BUN (mg/dL)	13.3 (8.6, 22.9)	13.55 ± 3.24
Creatinine (mg/dL)	0.9 (0.7, 1.2)	0.91 ± 0.11
CrCl (mL/min)	124.41 (86.57, 159.05)	124.55 ± 17.78

**Table 2:** Base model building steps.

Model	Description	nParameter	-2LL	AIC	△-2LL	△AIC
**Total cefprozil**						
Absorption model						
01	No T_lag_	7	1547.07	1561.07		
02 *	Add T_lag_	9	1328.15	1346.15	−218.92	−214.92
Residual error model						
02-01	Proportional	9	1328.15	1346.15		
02-02	Additive	9	1479.58	1497.58	151.42	151.42
02-03 *	Log additive	9	23.04	41.04	−1305.11	−1305.11
IIV model						
02-03-01	Remove IIV V	8	23.04	39.04	0.00	−2.00
02-03-02	Remove IIV Cl	8	69.66	85.66	46.62	44.62
02-03-03 *	Remove IIV K_a_	8	0.83	16.83	−22.21	−24.21
02-03-04	Remove IIV T_lag_	8	56.45	72.45	33.41	31.41
02-03-05	Remove IIV V, K_a_	7	217.49	231.49	194.45	190.45
***Cis*-cefprozil**						
Absorption model						
01	No T_lag_	7	1478.56	1492.56		
02 *	Add T_lag_	9	1266.71	1284.71	−211.84	−207.84
Residual error model						
02-01	Proportional	9	1266.71	1284.71		
02-02	Additive	9	1416.03	1434.03	149.32	149.32
02-03 *	Log additive	9	33.82	51.82	−1232.89	−1232.89
IIV model						
02-03-01	Remove IIV V	8	33.82	49.82	0.00	−2.00
02-03-02	Remove IIV Cl	8	81.03	97.03	47.21	45.21
02-03-03 *	Remove IIV K_a_	8	12.57	28.57	−21.25	−23.25
02-03-04	Remove IIV T_lag_	8	63.44	79.44	29.62	27.62
02-03-05	Remove IIV V, K_a_	7	224.58	238.58	190.76	186.76
***Trans*-cefprozil**						
Absorption model						
01	No T_lag_	7	−60.47	−46.47		
02 *	Add T_lag_	9	−251.70	−233.70	−191.23	−187.23
Residual error model						
02-01 *	Proportional	9	−251.70	−233.70		
02-02	Additive	9	−109.81	−91.81	141.89	141.89
02-03	Log additive	9	188.55	206.55	440.25	440.25
IIV model						
02-03-01 *	Remove IIV V	8	−251.70	−235.70	0.00	−2.00
02-03-02	Remove IIV Cl	8	−184.37	−168.37	67.33	65.33
02-03-03	Remove IIV K_a_	8	12.11	28.11	263.81	261.81
02-03-04	Remove IIV T_lag_	8	−202.32	−186.32	49.39	47.39

* Selected model.

**Table 3:** Stepwise search for covariates.

Model	OFV	△OFV
**Total cefprozil**		
Base model	0.829	
Total protein on clearance	0.693	−0.136
Albumin on clearance	0.342	−0.487
CrCl on clearance *	−6.502	−7.331
Weight on volume	−2.075	−2.904
BSA on clearance	−1.585	−2.413
***Cis*-cefprozil**		
Base model	12.568	
Total protein on clearance	12.385	−0.184
Albumin on clearance	11.958	−0.611
CrCl on clearance *	4.901	−7.668
Weight on volume	9.956	−2.612
BSA on clearance	9.287	−3.282
***Trans*-cefprozil**		
Base model *	−251.70	
Total protein on clearance	−250.41	1.292
Albumin on clearance	−254.46	−2.763
CrCl on clearance	−248.54	3.162
BSA on clearance	−252.38	−0.676

* Final model.

**Table 4:** Population pharmacokinetic parameters for cefprozil in base and final models.

Parameter	Estimate	SE	RSE (%)	Shrinkage (%)
**Total cefprozil**				
Base model				
tvV (mL)	14,707.50	1200.54	8.16	
tvCl (mL/h)	17,189.00	451.80	2.63	
tvTlag (h)	0.352	0.013	3.73	
tvKa (1/h)	0.432	0.006	1.28	
ω^2^_V_	0.123	0.027	21.62	7.03
ω^2^_Cl_	0.018	0.005	26.15	9.05
ω^2^_Tlag_	0.020	0.007	32.05	21.43
σ (μg/mL)	0.190	0.017	9.03	
Final model				
tvV (mL)	14,713.10	1200.37	8.16	
tvCl (mL/h)	17,226.20	405.12	2.35	
tvTlag (h)	0.351	0.013	3.74	
tvKa (1/h)	0.432	0.006	1.28	
dCldCrCl	0.003	0.002	56.16	
ω^2^_V_	0.124	0.027	21.49	7.08
ω^2^_Cl_	0.016	0.004	25.11	10.39
ω^2^_Tlag_	0.021	0.007	31.96	21.39
σ (μg/mL)	0.189	0.017	9.02	
***Cis*-cefprozil**				
Base model				
tvV (mL)	14,307.00	1182.54	8.27	
tvCl (mL/h)	17,111.30	456.71	2.67	
tvTlag (h)	0.352	0.014	3.91	
tvKa (1/h)	0.429	0.006	1.32	
ω^2^_V_	0.127	0.027	21.66	7.39
ω^2^_Cl_	0.019	0.005	26.58	9.02
ω^2^_Tlag_	0.021	0.007	33.09	21.28
σ (μg/mL)	0.193	0.016	8.41	
Final model				
tvV (mL)	14,308.10	1184.69	8.28	
tvCl (mL/h)	17,150.70	406.51	2.37	
tvTlag (h)	0.352	0.014	3.92	
tvKa (1/h)	0.429	0.006	1.33	
dCldCrCl	0.003	0.002	51.87	
ω^2^_V_	0.127	0.027	21.53	7.32
ω^2^_Cl_	0.016	0.004	24.57	10.48
ω^2^_Tlag_	0.021	0.007	33.25	21.23
σ (μg/mL)	0.193	0.016	8.40	
***Trans*-cefprozil**				
Final model				
tvV (mL)	34,617.50	1997.67	5.77	
tvCl (mL/h)	17,701.80	598.23	3.38	
tvTlag (h)	0.352	0.018	5.09	
tvKa (1/h)	0.829	0.091	10.98	
ω^2^_Cl_	0.017	0.006	37.04	9.28
ω^2^_Tlag_	0.094	0.042	44.62	12.06
ω^2^_ka_	0.076	0.025	32.56	10.95
σ	0.232	0.015	6.61	

**Table 5:** Population pharmacokinetic parameter estimates for cefprozil and bootstrap validation.

Parameter	Final Model		Bootstrap	
	Estimate	95% CI	Median	95% CI
**Total cefprozil**				
tvV (mL)	14,713.10	12,352.34–17,073.86	14,777.30	12,595.74–17,799.95
tvCl (mL/h)	17,226.20	16,429.46–18,022.94	17,233.30	16,418.17–18,051.63
tvTlag (h)	0.351	0.326–0.377	0.352	0.330–0.373
tvKa (1/h)	0.432	0.421–0.443	0.433	0.422–0.446
dCldCrCl	2.87 × 10^−3^	−3.00 × 10^−4^–6.04 × 10^−3^	2.87 × 10^−3^	−3.08 × 10^−4^–6.27 × 10^−3^
ω^2^_V_	0.124	0.072–0.176	0.121	0.065–0.177
ω^2^_Cl_	0.016	0.006–0.025	0.014	0.006–0.021
ω^2^_Tlag_	0.021	0.008–0.033	0.020	0.006–0.034
σ (μg/mL)	0.189	0.156–0.223	0.189	0.157–0.222
***Cis*-cefprozil**				
tvV (mL)	143,08.10	11,978.18–16,638.02	14,406.04	12,334.79–17,164.45
tvCl (mL/h)	17,150.70	16,351.22–17,950.18	17,191.88	16,488.92–17,926.22
tvTlag (h)	0.352	0.325–0.379	0.354	0.323–0.378
tvKa (1/h)	0.429	0.418–0.440	0.429	0.420–0.442
dCldCrCl	3.04 × 10^−3^	−6.10 × 10^−5^–6.15 × 10^−3^	3.06 × 10^−3^	7.24 × 10^−5^–6.04 × 10^−3^
ω^2^_V_	0.127	0.073–0.181	0.122	0.066–0.179
ω^2^_Cl_	0.016	0.006–0.026	0.015	0.007–0.023
ω^2^_Tlag_	0.021	0.007–0.034	0.021	0.006–0.035
σ (μg/mL)	0.193	0.161–0.225	0.193	0.163–0.225
***Trans*-cefprozil**				
tvV (mL)	34,617.50	30,688.77–38,546.24	34,360.90	22,079.72–38,114.42
tvCl (mL/h)	17,701.80	16,525.29–18,878.31	17,636.80	16,492.77–18,807.66
tvTlag (h)	0.352	0.317–0.388	0.356	0.317–0.393
tvKa (1/h)	0.829	0.650–1.008	0.828	0.510–1.010
ω^2^_Cl_	0.017	0.005–0.029	0.018	0.001–0.035
ω^2^_Tlag_	0.094	0.012–0.177	0.108	0.007–0.222
ω^2^_ka_	0.076	0.028–0.125	0.071	0.014–0.128
σ	0.232	0.202–0.262	0.228	0.196–0.266
